# Evaluation of digital dispense-assisted broth microdilution antimicrobial susceptibility testing for *Pseudomonas aeruginosa* isolates

**DOI:** 10.1038/s41598-021-88423-0

**Published:** 2021-04-28

**Authors:** Shawn T. Clark, Patrick J. Stapleton, Pauline W. Wang, Yvonne C. W. Yau, Valerie J. Waters, David M. Hwang, David S. Guttman

**Affiliations:** 1 Department of Cell & Systems Biology, University of Toronto, Toronto, ON Canada; 2 Department of Laboratory Medicine and Pathobiology, University of Toronto, Toronto, ON Canada; 3 Department of Pediatric Laboratory Medicine, Division of Microbiology, The Hospital for Sick Children, Toronto, ON Canada; 4 Centre for the Analysis of Genome Evolution and Function, University of Toronto, Toronto, ON Canada; 5 Department of Pediatrics, Division of Infectious Diseases, The Hospital for Sick Children, Toronto, ON Canada; 6 Department of Laboratory Medicine & Molecular Diagnostics, Sunnybrook Health Sciences Centre, Toronto, Canada

**Keywords:** Antimicrobial resistance, Antimicrobials, Clinical microbiology

## Abstract

Antimicrobial susceptibility testing (AST) is essential for detecting resistance in *Pseudomonas aeruginosa* and other bacterial pathogens. Here we evaluated the performance of broth microdilution (BMD) panels created using a semi-automated liquid handler, the D300e Digital Dispenser (Tecan Group Ltd., CH) that relies on inkjet printing technology. Microtitre panels (96-well) containing nine twofold dilutions of 12 antimicrobials from five classes (β-lactams, β-lactam/β-lactamase inhibitors, aminoglycosides, fluoroquinolones, polymyxins) were prepared in parallel using the D300e Digital Dispenser and standard methods described by CLSI/ISO. To assess performance, panels were challenged with three well characterized quality control organisms and 100 clinical *P. aeruginosa* isolates. Traditional agreement and error measures were used for evaluation. Essential (EA) and categorical (CA) agreements were 92.7% and 98.0% respectively for *P. aeruginosa* isolates with evaluable on-scale results. The majority of minor errors that fell outside acceptable EA parameters (≥ ± 1 dilution, 1.9%) were seen with aztreonam (5%) and ceftazidime (4%), however all antimicrobials displayed acceptable performance in this situation. Differences in MIC were often log_2_ dilution lower for D300e dispensed panels. Major and very major errors were noted for aztreonam (2.6%) and cefepime (1.7%) respectively. The variable performance of D300e panels suggests that further testing is required to confirm their diagnostic utility for *P. aeruginosa*.

## Introduction

Antimicrobial susceptibility testing (AST) and reporting are critical factors involved in the treatment of many bacterial infections. In principle, AST is designed to provide timely, accurate and reliable results that allow physicians to tailor antimicrobial therapies to ensure optimal coverage for the pathogen(s) believed to be involved in an infectious process based on their in vitro phenotypic properties. However, in practice, our ability to accurately perform AST and detect resistance to some antimicrobials for many organisms is far from ideal^1^.

Broth microdilution (BMD) is the preferred method to determine the minimum inhibitory concentrations (MIC) of *P. aeruginosa* and many other pathogens by AST, as recommended by the Clinical and Laboratory Standards Institute (CLSI), the European Committee on Antimicrobial Susceptibility Testing and the International Organization for Standardization^2,3^. Clinical microbiology laboratories perform AST by a variety of MIC-based and MIC surrogate approaches that differ in speed, cost and complexity, including commercial automated systems and disk or gradient diffusion methods^4^. Several recent studies have suggested that AST by BMD could be performed in a fully customizable, routine manner using a liquid handling device that functions through inkjet printing technology^5–9^. The D300e Digital Dispenser (Tecan Group Ltd., CH) is a benchtop liquid handler that has a small laboratory footprint and is designed to dispense minute quantities (nanolitres to picolitres) of biological solutions into the wells or microtitre plates, with applications that have ranged from assessing the activity and cytotoxicity of novel compounds^10^, vaccine potency^11^, analytical quality control practices^12^ to AST^5–9^.

When used for AST panel creation, the D300e instrument dispenses variable volumes of a fixed antimicrobial stock solution into the test wells of microtitre plates to achieve concentrations equivalent to those measured in a traditional twofold dilution series. The performance of D300e-created panels for AST by BMD has been examined in collections of *Enterobacterales* isolates, where MICs and performance characteristics have been in agreement with the reference BMD method described by CLSI^5–9^. To-date, few errors in classification have been reported for several antimicrobials, including cefepime^5,6,9^. A recent pilot study led to the instrument being used to create panels for specialized AST across several public health laboratories in the United States^9^. As D300e-assisted testing has been focused primarily on members of the *Enterobacterales*, it is unclear whether D300e created panels could also be used for AST of non-*Enterobacterales* organisms with more complex mechanisms of resistance such as *P. aeruginosa*, as these isolates have not been included in published challenge panels. The objective of this study was to evaluate the performance of *P. aeruginosa*-focused AST panels created by the D300e Digital Dispenser containing 12 antimicrobials with varying antipseudomonal activity, as compared to reference BMD (rBMD) methods.

## Materials and methods

### Bacterial isolates and culture conditions

All bacterial isolates, frozen in 20% (v/v) glycerol at − 80 °C, were sub-cultured twice on non-selective Luria Bertani (LB) agar and grown at 35 °C ± 2 °C for 24 h prior to AST. The *Escherichia coli* ATCC 25922, *Escherichia coli* ATCC 35218 and *Pseudomonas aeruginosa* ATCC 27853 strains were selected for quality control. One hundred clinical *P. aeruginosa* isolates were selected for assay verification as described in the CLSI M52 document^13^. The *P. aeruginosa* isolates has been collected and stored following routine microbiological analysis between 1999 and 2017. Isolates were not tested for clonality but were selected from unique patients where possible. Sputum isolates were cultured from cystic fibrosis patients attending clinic at the Hospital for Sick Children (n = 25) or those with (n = 25) or without (n = 10) cystic fibrosis attending clinics at St. Michael’s Hospital (n = 25) (Toronto, Canada). Those from blood or other sterile sites, urine or rectal swab isolates (n = 30) were collected from children at the Hospital for Sick Children (Toronto, Canada). The remaining isolates (n = 10) were collected from miscellaneous body sites (e.g. ear, eye). The isolates reflected a diversity of phenotypes that may be encountered in a diagnostic laboratory, with different colonial morphologies and anatomical sources of isolation included (Table S1).

### Preparation of growth media and antimicrobials

Cation-adjusted Mueller–Hinton II broth (CAMHB) (BD Diagnostics, USA) was prepared according to the manufacturer’s recommendation. Twelve antimicrobial agents from five classes (β-lactams, β-lactam/β-lactamase inhibitor combinations, aminoglycosides, polymyxins, fluoroquinolones) were included in the test panels. Amikacin (AMK), aztreonam (ATM), cefepime (FEP), ciprofloxacin (CIP), colistin (CST), gentamicin (GEN), levofloxacin (LVX), tobramycin (TOB) were purchased from Alfa Aesar (MA, USA), meropenem (MEM) was purchased from US Biological (MA, USA), ceftazidime, piperacillin and tazobactam (TZP) were purchased from Sigma Aldrich (ON, Canada) and Alfa Aesar (MA, USA) respectively, and imipenem (IPM) was purchased from Chem-Impex International Inc (IL, USA). Antimicrobial stock solutions were prepared by dissolving each antimicrobial powder in CLSI-recommended solvents^3,14^ and storing as aliquots at − 20 °C. Working solutions were diluted in CAMHB from the corresponding stock on the day of testing.

### Preparation of broth microdilution panels

All panels were prepared on-demand in 96-well, non-tissue culture treated microplates (BioMart Canada, ON, Canada) as described in the CLSI M07^14^. Each new batch of panels was tested with the *E. coli* ATCC 25922, *E. coli* ATCC 35218 and *P. aeruginosa* ATCC 27853 strains to ensure appropriate QC^3^. The same lot of antimicrobial powders, working solutions and CAMHB, as well as 0.5 McFarland suspensions were used to inoculate both rBMD and D300e BMD panels in parallel on the same day. Spent antimicrobial aliquots were discarded after a single use. Panels for AST of QC organisms created by D300e and BMD contained nine concentrations of the 12 test antimicrobials in a twofold dilution series while panels for *P. aeruginosa* isolates contained the same antimicrobials at higher concentrations (Table 1).

**Table 1:** Antimicrobials contained in D300e and rBMD panels.

Antimicrobial	Quality control panels (µg/mL)	Clinical isolate panels (µg/mL)
**Aminoglycosides**		
Amikacin	0.03 to 8	1 to 256
Gentamicin	0.03 to 8	0.5 to 128
Tobramycin	0.008 to 2	0.5 to 128
**β-lactams**		
Aztreonam	0.06 to 16	1 to 256
Cefepime	0.03 to 8	0.5 to 128
Ceftazidime	0.03 to 8	1 to 256
Imipenem	0.02 to 4	0.25 to 64
Meropenem	0.008 to 2	0.25 to 64
Piperacillin-tazobactam	0.06/4 to 16/4	1/4 to 256/4
**Fluoroquinolones**		
Ciprofloxacin	0.008 to 2	0.12 to 32
Levofloxacin	0.03 to 8	0.25 to 64
**Polymyxin**		
Colistin	0.03 to 8	0.12 to 32

### Preparation of digitally-dispensed broth microdilution panels

Automated dispensing by the D300e was programmed using the D300e Control software (v 3.3.2) (HP Development Company L.P., USA). Each microtitre plate was formatted using the Titration feature, which determined the input volume of an antimicrobial working stock and the amount dispensed per well to achieve the desired final concentration over a logarithmic distribution. Antimicrobial solutions were dispensed using T8 + Dispensehead Cassettes (Tecan Group Ltd., CH) with 8 single-use dispenseheads (10 µL maximum dispense volume). Final antimicrobial dispense volumes ranged from 6.8 µL (for 256 µg/mL wells) to 7.8 × 10^–4^ µL (for 0.008 µg/mL wells) per well. To assist with digital dispensing, a solution of 0.3% polysorbate P-20 (Sigma Aldrich, ON, Canada) surfactant was incorporated in each antimicrobial working solution as previously described^5^. The final concentration of P-20 post-dispense was negligible, ranging from 0.019% (for 256 µg/mL wells) to 6.2 × 10^–7^% (for 0.008 µg/mL wells) per well. Pre-test pilot studies indicated that the P-20 surfactant did not inhibit or impair the growth rate of any QC strains across the test range, as determined by optical density measurements at OD_600_ (Data not shown).

### Antimicrobial susceptibility testing

AST was performed in accordance with CLSI M07-A10 guidelines for BMD^14^. Briefly, bacterial cell suspensions equivalent to a 0.5 McFarland standard were prepared by emulsifying colonies from fresh sub-cultures in 0.9% (w/v) NaCl and verified using an Oxoid Turbidimeter (Oxoid Canada, ON, CA). The same 0.5 McFarland suspension was used to create the respective starting inoculum for the D300e or rBMD methods on the same day and was diluted in CAMHB to an appropriate intermediate concentration in order to achieve a final inoculum density of 5 × 10^5^ CFU/mL per well (100 µL), as confirmed by colony counts. The diluted inoculum was added to each plate with a multichannel pipette and test panels were incubated statically at 35ºC ± 2 °C for 20 to 24 h. The MIC for each antimicrobial-organism combination was assessed visually against a black background by a single reader and reported as the lowest concentration of the agent that completely inhibited isolate growth. Isolates were numerically coded to blind the reader to the identity of the test isolates. The corresponding MICs for each antimicrobial/organism combination were determined using CLSI 2020 breakpoints^3^.

### Reproducibility testing

The reproducibility of the D300e BMD approach was assessed against the rBMD method using a 15 replicate quality control strategy. The *E. coli* ATCC 25922 (β-lactamase negative), *E. coli* ATCC 35218 (TEM-1 β-lactamase) and *P. aeruginosa* ATCC 27853 (inducible AmpC β-lactamase) strains were tested in triplicate for five consecutive days by both methods. A separate 0.5 McFarland cell suspension was used to inoculate each biological replicate.

### Data analysis

The accuracy and reproducibility of the D300e panels were evaluated using standard error measures^15^. The proportion of D300e MICs that were within acceptable CLSI QC ranges was determined by comparing the log_2_ difference for each on-scale D300e measurement (an MIC within the concentrations tested) to its corresponding modal rBMD MIC for each QC organism-antimicrobial combination. Evaluable essential agreement (EEA) was calculated as the % of on-scale results that were within a 1log_2_ dilution for D300e and rBMD panels. Overall essential (EA) and categorical agreement (CA) were calculated using the total number of MIC measurements and the categorical matches respectively, across the 5-day period. Acceptance criteria for precision essential and categorical agreement were ≥ 90%.

For the clinical *P. aeruginosa* strain panel, EEA, EA and CA were used to compare the agreement between methods. Variations between methods were classified as very major errors (VME), isolates whose MICs were reported as susceptible by D300e BMD and resistant by rBMD, major errors (ME), those reported as resistant by D300e BMD and susceptible by rBMD, or minor errors (mE) with an isolate testing as intermediate in one method, while testing as susceptible or resistant in the other (only D300e MICs > 1log_2_ dilution of rBMD), and applied only when all three interpretative categories are available^3,13,15^. To better reflect error rates, we also calculated mE for MICs outside EA for any measurement that was ≥ 2log_2_ dilutions above or below the rBMD MIC. The total numbers of resistant and susceptible *P. aeruginosa* isolates for each antimicrobial agent were used as the denominator when calculating the VME and ME respectively. Acceptance criteria were defined as ≤ 10% for mE, ≤ 3% for ME and ≤ 1.5% for VME^15^. The full AST panels were repeated in triplicate by both test methods for any isolates that displayed either ME or VME for any antimicrobial in the panels. Errors resolved upon repeat testing were treated as previously described^16^. The 95% confidence intervals, distribution of MICs, Bland–Altman analysis and correlations in MIC between methods (Spearman correlation coefficient) were determined in GraphPad Prism v 8.4.2 (CA, USA).

## Results

### Reproducibility of D300e dispensed panels in quality control screens

Fifteen QC panels created by the D300e Digital Dispenser were compared to traditional rBMD using the CLSI-recommended *E. coli* ATCC 25922, *E. coli* ATCC 35218, and *P. aeruginosa* ATCC 27853 control strains. Of the 1080 MIC measurements collected, 79.1% of D300e and 81.2% of rBMD measurements were on-scale and used to evaluate EA (Table 2). The majority of on-scale D300e MICs (98.9%) were within a 1log_2_ dilution of the modal MIC from comparable rBMD testing and in high evaluable essential agreement (Table 2). The percent of on-scale results within acceptable QC ranges stated by CLSI was 90.0% (range: 82.5 to 96.1). The overall EA which included both on- and off-scale measurements was acceptable at 96.5%, however variability was noted among the test organisms/antimicrobial combinations (Table 2). Individually all control strains had acceptable EA, with an average of 94.4% (range: 66.7–100.0%) for the *E. coli* ATCC 29522 strain, 95.0% (range: 60.0–100.0%) for *E. coli* ATCC 35218, and 100.0% (range: 100.0%) for *P. aeruginosa* ATCC 27853 (Table 2). Measurements for LVX for *E.coli* ATCC 25922, FEP and TZP with *E. coli* ATCC 35218 were off-scale and not included in the EEA analysis. Poor overall EA and EEA were observed with CST for both *E. coli* ATCC 25922 and *P. aeruginosa* ATCC 27853 strains in D300e created panels. While these data were in overall CA, individual CST D300e measurements were often spread over a ± 3 log_2_ dilution range from the corresponding rBMD measurements. In contrast, the MICs measured for the *P. aeruginosa* ATCC 27853 strain were highly reproducible in all tests (Table 2). Despite the discordance in CST QC (Table 2), the D300e dispensed panels had excellent CA with the rBMD method for all strains, with no categorical discrepancies noted for any antimicrobial (100% CA for all antimicrobials/QC strain pairings).

**Table 2:** Reproducibility of D300e dispensed AST panels assessed with 3 quality control strains.

Antimicrobial	Log_2_ difference in MIC from rBMD modal MIC^a^						Percent of on-scale results within acceptable QC range (%)^b,c^			Evaluable essential agreement (%, 95% CI)	Overall essential agreement (%, 95% CI)
	≤ − 3	− 2	− 1	0	1	≥ 2	*P. aeruginosa* ATCC 27853	*E. coli* ATCC 35218	*E. coli* ATCC 2922		
AMK	0	0	10	27	8	0	100.0	n/a	100.0	100.0	100.0
ATM	0	0	2	37	6	0	100.0	73.3	80.0	84.4	100.0
FEP	0	0	0	24	1	0	100.0	–^d^	90.0	95.0	100.0
CAZ	0	0	10	29	3	0	100.0	n/a	100.0	100.0	93.3
CIP	0	0	0	14	3	0	100.0	n/a	100.0	100.0	95.6
CST	1	1	16	18	0	0	60.0	n/a	20.0	40.0	75.6
GEN	0	0	8	44	9	1	93.3	n/a	100.0	96.7	100.0
IPM	0	0	0	45	0	0	100.0	100.0	100.0	100.0	100.0
LVX	0	0	0	15	0	0	100.0	n/a	–^d^	100.0	97.8
MEM	0	0	0	37	6	0	100.0	100.0	100.0	100.0	100.0
TZP	0	1	10	16	3	0	100.0	–^d^	100.0	100.0	97.8
TOB	0	0	6	34	4	1	100.0	n/a	100.0	100.0	97.8
Total (n,%, 95% CI)	1 (0.2)	2 (0.4)	62 (13.8)	340 (75.6)	43 (9.6)	2 (0.4)	96.1 (88.7–100.0)	91.1 (61.7–100.0)	82.5 (73.8–100.0)	93.0 (82.0–100.0)	96.5 (92.1–100.0)

^a^Modal MIC values were calculated as the mode of all on-scale rBMD measurements for each antimicrobial-organism combination.

^b^Quality control MIC ranges for each control organism were determined using the CLSI M100 MIC ranges for nonfastidious QC for both non-β-lactam combination and β-lactam combination agents^3^.

^c^Organisms without QC ranges provided by CLSI for a particular antimicrobial are listed as n/a.

^d^Organism-antimicrobial combinations that did not generate on-scale MIC results within the QC range are listed as –.

### Performance of D300e panels with clinical *P. aeruginosa* isolates

Isolates from the verification panel (Figure S1) were found to display a wide array of antimicrobial-associated phenotypes, with resistance to any of the 12 antimicrobial agents being documented for at least one isolate (as determined by rBMD) (Table S1, Table S2). The most active antipseudomonal agents were CST, MEM and TZP (with 98.0, 69.0 and 72.0% of isolates being susceptible, respectively), while GEN was the least active (42.0% of isolates displaying susceptibility) (Table S2).

**Figure 1:**
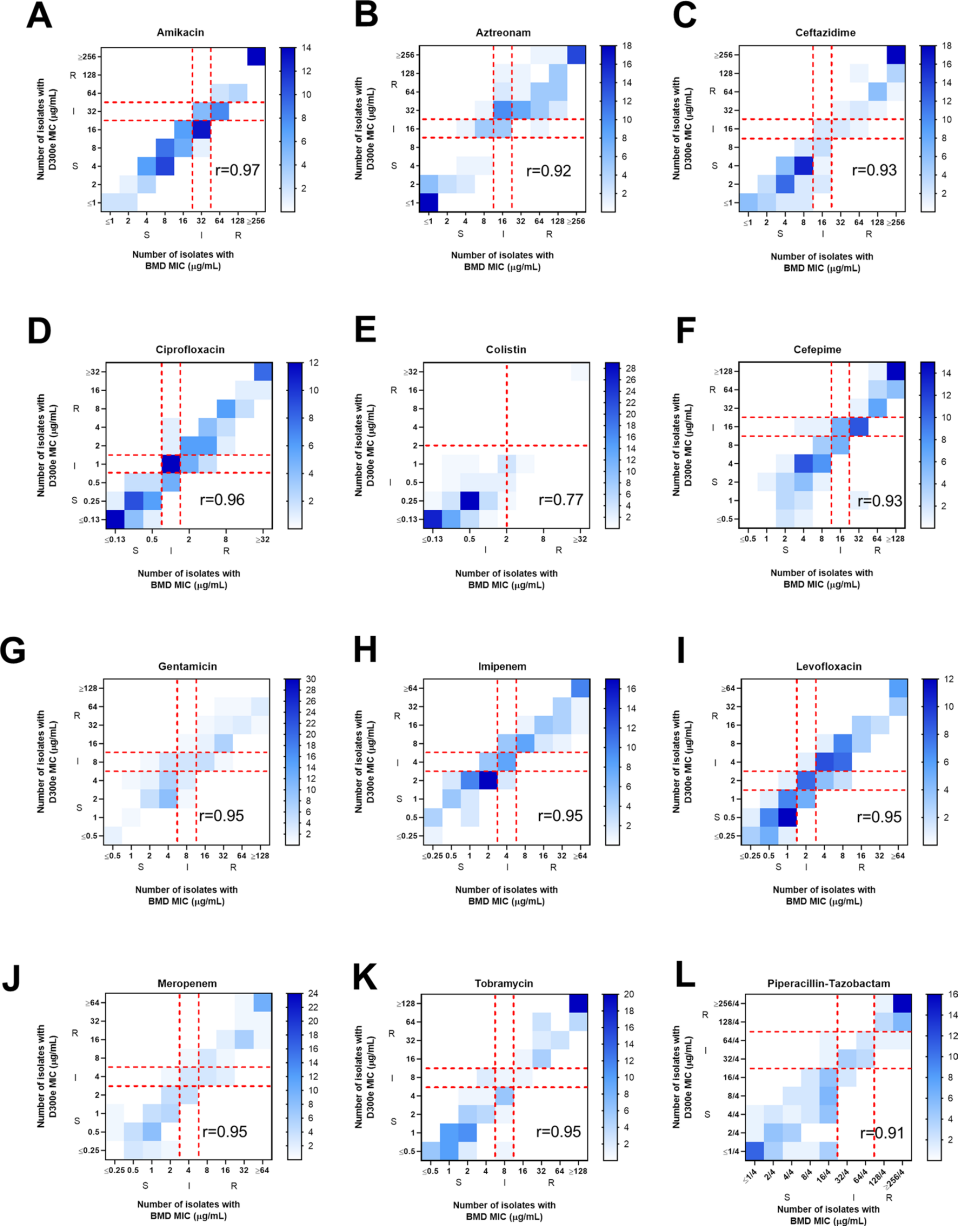
Scattergrams of the D300e and rBMD MIC comparisons (µg/mL). (**A**) amikacin, (**B**) aztreonam, (**C**) ceftazidime, (**D**) ciprofloxacin, (**E**) colistin, (**F**) cefepime, (**G**) gentamicin, (**H**) imipenem, (**I**) levofloxacin, (**J**) meropenem, (**K**) tobramycin, (**L**) piperacillin-tazobactam.

**Figure 2:**
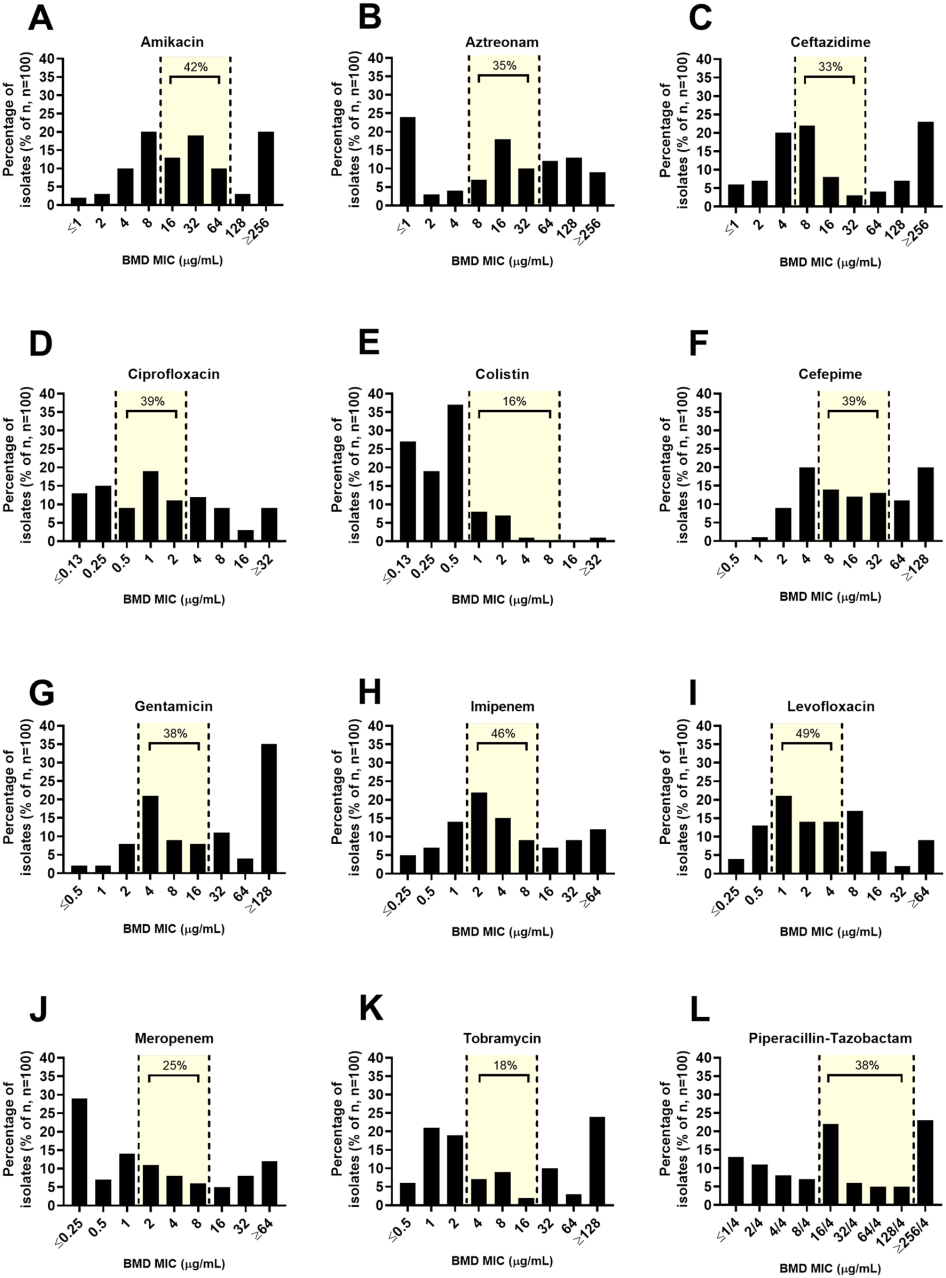
Distribution of MICs for the 100 *P. aeruginosa* isolates. The frequencies of different MICs for each of the antimicrobials tested were tabulated as a percentage of isolates (%) with a particular MIC value (µg/mL) as measured by rBMD for: (**A**) amikacin, (**B**) aztreonam, (**C**) ceftazidime, (**D**) ciprofloxacin, (**E**) colistin, (**F**) cefepime, (**G**) gentamicin, (**H**) imipenem, (**I**) levofloxacin, (**J**) meropenem, (**K**) tobramycin, (**L**) piperacillin-tazobactam. Yellow shading indicates the 2020 CLSI rBMD interpretive range for each of the antibiotics. Hatched borders indicate the lower and upper distinctions for MIC interpretation, where isolates were deemed susceptible (lowest concentration and below), intermediate (middle concentration) or resistant (highest concentration or above).

Eighty-one percent of the D300e MICs were on-scale and within 1log_2_ dilution of the corresponding rBMD value. In general, MICs from D300e dispensed panels correlated well with rBMD panels, yielding an average Spearman correlation coefficient of 0.93 (range: 0.77 to 0.97, n = 1200 per panel type) (Fig. 1). The D300e and rBMD methods were found to have the greatest variability in mean MIC for AMK, CAZ and TZP (Figure S2). There was an average overall EA and EEA of 92.7% for all antibiotics tested, with the highest EA and EEA for the aminoglycosides and fluoroquinolones (97.0 and 96.0% respectively) and the lowest EA for CST (84.0%) (Table 3). Despite the overall high agreement, measurements for only eight of the 12 antimicrobials were in acceptable (≥ 90%) overall EA and EEA. Some β-lactams had the greatest variation in measurement, with low EA and EEA for ATM, CAZ and TZP. In contrast, the carbapenems MEM and IPM were both acceptable^13,15^. We noted that MICs that differed between methods (i.e. the same MIC in µg/mL was not reported in both methods) were typically 1 log_2_ dilution lower in D300e panels, which accounted for 35.8% of the total measurements. When examining the distribution of MICs among isolates, rBMD MICs were often at breakpoint concentrations for susceptible, intermediate and resistant as denoted by CLSI (range: 18.0 to 49.0%) (Fig. 2). This was particularly true of MICs measured for AMK, IPM and LVX, with 42.0, 46.0 and 49.0% of isolates tested having an MIC that fell within the 3 log_2_ dilution breakpoint range.

**Table 3:** Difference in MICs between D300e and rBMD panels for *P. aeruginosa* isolates.

Antimicrobial	No. of dilutions from rBMD result^a^							Overall EA (%)	# in EEA^b^	EEA (%)	Overall CA (%)^c^	CA outside EA (%)^d^	mE (%)	mE outside EA (%)^e^	ME (%) (# of S)^g^	VME (%) (# of R)^h^
	≤ − 3	− 2	− 1	0	1	2	≥ 3									
AMK	0	1	52	47	0	0	0	98.0	85	98.8	78.0	99.0	22.0	1.0	0 (48)	0 (34)
ATM	0	4	15	51	23	5	2	89.0	94	88.3	79.0	94.0	20.0	5.0	2.6 (38)	0 (44)
FEP	1	4	46	45	4	0	0	95.0	87	94.2	80.0	99.0	19.0	1.0	0 (44)	1.7 (56)
CAZ	4	8	49	38	1	0	0	88.0	83	86.6	91.0	96.0	9.0	4.0	0 (55)	0 (37)
CIP	0	4	31	60	4	1	0	95.0	91	94.5	86.0	97.0	14.0	3.0	0 (37)	0 (44)
CST	2	13	50	32	2	1	0	84.0	98	83.7	99.0	99.0	1.0	1.0	n/a^f^	n/a^f^
GEN	0	3	32	55	9	1	0	96.0	72	98.6	85.0	98.0	15.0	2.0	0 (34)	0 (58)
IPM	0	1	12	54	30	3	0	96.0	92	95.6	87.0	100.0	13.0	0.0	0 (48)	0 (37)
LVX	0	3	42	50	5	0	0	97.0	91	96.8	87.0	98.0	13.0	2.0	0 (38)	0 (48)
MEM	0	7	31	58	3	1	0	92.0	92	91.3	89.0	100.0	11.0	0.0	0 (61)	0 (31)
TZP	5	6	28	49	9	3	0	86.0	83	85.5	94.0	98.0	6.0	2.0	0 (61)	0 (28)
TOB	1	3	42	48	6	0	0	96.0	80	98.7	89.0	98.0	11.0	2.0	0 (52)	0 (39)
Total (n, %, 95% CI)	13 (1.08)	57 (4.75)	430 (35.8)	587 (48.9)	96 (8.0)	15 (1.25)	2 (0.17)	92.7 (89.6–95.7)	87.3	92.7 (89.2–92.7)	87.0 (82.9–90.9)	98.0 (96.9–99.1)	12.8	1.91	0.22	0.14

^a^The total number of D300e MICs (on- and off-scale) that were compared to equivalent rBMD MIC.

^b^Calculated using the evaluable on-scale MICs that were within 1log2 dilution between D300e and rBMD dispensed panels.

^c^Agreement of S,I,R categories between D300e and rBMD panels determined using CLSI 2020 criteria among 1,200 MIC measurements per method.

^d^Calculated by identifying the number of strains where CA differences are ≥  ± 2log_2_ dilutions from the corresponding rBMD MIC.

^e^Calculated by identifying the number of minor errors in strains where D300e and rBMD MICs were ≥  ± 2log_2_ dilutions apart.

^f^There are only I or R breakpoints provided for CST by CLSI^3^.

^g^The number of susceptible isolates was used as the denominator in the calculation of ME.

^h^The number of resistant isolates was used as the denominator in the calculation of VME.

The clustering of isolate MICs among the interpretive breakpoints was also reflected in the overall categorical agreement (CA) parameters. The average overall CA between D300e and rBMD panels was 87.0% (82.9–90.9, 95% CI), with the lowest CA being reported for AMK and ATM (at 78.0 and 79.0% CA respectively). This led to only 3 of the 12 antimicrobials tested meeting acceptable ≥ 90% CA criteria. When accounting for the high proportion of CA discrepancies resulting from MIC shifts occurring within the acceptable ± 1log_2_ dilution performance of BMD tests (measurements in EA), we noted significant CA improvement, reaching ≥ 90% CA for all antimicrobial-organism combinations (Table 3). Rates of mE were highest for AMK, ATM and FEP (Table 3), with 60.0% of all mE being between susceptible and intermediate interpretations. Only CAZ, CST and TZP met mE < 10% threshold. Similar to CA discrepancies, the majority of mEs remained within acceptable 1 log_2_ dilution EA for BMD methods (Table 3). When examining only mE outside EA, error rates dramatically decreased to acceptable levels for both mE and ME (< 10% for mE, < 3% ME) for all antimicrobial-organism combinations. A high VME rate was identified for FEP (1.7%). Repeat testing of two *P. aeruginosa* isolates with VME or ME for ATM or FEP in triplicate (as described^16^) did not resolve these errors (Table S3). These discrepancies were in separate, unrelated isolates sourced from wound or eye swabs. There was no difference in error rate based on colony phenotype; however colonies from two of the three isolates with VME or ME were non-mucoid.

## Discussion

It is important that AST methods provide a high degree of confidence when testing *P. aeruginosa* and other bacterial pathogens as these results can impact patient care. The D300e dispensed BMD panels created in this study were challenged with a diverse array of *P. aeruginosa* isolates selected to represent strains from different specimen types, those with unique morphologic properties and/or resistance mechanisms. Under these conditions, the D300e panels did not perform as expected, displaying a relatively high minor error rate and because of this, did not meet acceptance criteria for overall EA or CA for several antimicrobials tested. Eight of the 12 antimicrobial agents met acceptable EA (≥ 90%), with several β-lactams and CST displaying more variable MIC patterns between methods. A similar trend was noted with categorical agreement, with only three antibiotics meeting acceptable performance criteria (≥ 90%). Many of the mE identified were within EA and the acceptable performance of a BMD test (a 1 log_2_ dilution of the rBMD MIC value), with the D300e dispensed panels often having the lower MIC value for some antimicrobial agents, which was reflected in the discordance between MIC_50_ and MIC_90_ values between methods. The D300e panels performed well with CAZ and TZP, which had the lowest minor error rates among all antibiotics tested, as well as MEM and TOB which had good EA, EEA and CA and are important antipseudomonal agents. The performance of TZP in particular was an improvement over previously published ME and VME error rates for TZP testing, which have ranged from 4.8 to 22.0% depending on the system used for testing^1,17,18^ and are in agreement with those reported recently for newly reformulated TZP ETest strips^19^.

Two of the antimicrobial agents tested, ATM and FEP, had ME and VME that were unresolved upon repeat testing of two *P. aeruginosa* isolates. Previous comparisons of AST methods to detect β-lactam resistant *P. aeruginosa* strains noted similar challenges with ATM and FEP when tested by either disk diffusion^20^ or the VITEK 2 automated method^17,21^. The poor QC performance and EA of CST with clinical isolates was not surprising as it is an antimicrobial for which MIC testing is notoriously difficult^22,23^. Similar to earlier D300e tests by Smith and Kirby, in our hands, the D300e dispensed panels did not improve the ability to detect CST resistance patterns among the isolates tested. It is possible that this was an artifact of either the limited number of resistant isolates (denominator for VME calculation) that were tested by BMD from our region or the P20 surfactant used. A similar surfactant, polysorbate 80 (P-80) has been shown to reduce CST MICs at a concentration of 0.002% in organisms with low MICs, such as *E. coli*^22^. In our D300e dispensed QC plates, concentrations of the alternative surfactant P-20 were negligible (10^–7^% final concentration) at the lower end of the MIC range, but were above 0.002% in plates with pseudomonal-specific concentrations.

There are several factors that may explain the performance of the D300e panels examined in this study. The isolates used herein displayed elevated rates of resistance and a non-wild-type MIC distribution. Many isolates had MICs at interpretive breakpoints which could shift CA with slight differences in MICs between methods, and were reflected in the increased categorical error rates observed. While the error rates noted in the current study suggest that the D300e dispensed panels did not meet acceptable criteria, these criteria are primarily applied to less challenging wild type populations with lower MIC values. Given that reduced susceptibility to some antimicrobials has been observed among *P. aeruginosa* isolates circulating in certain clinical settings (e.g., intensive care units), isolates tested in this study were chosen to reflect these changing dynamics^24^. New AST approaches must be compared to a gold standard, which is often rBMD, a method with a similar amount of user input as D300e approaches, when performed as described by CLSI^14^. It appeared that many of the discrepancies identified in this study may be related to the randomness of MIC determinations and was not specific to the drug dispensing method. The high EA coupled with the knowledge that BMD tests have a 1-dilution error rate suggests that the D300e dispensed panels may still be suitable for selective use with certain antimicrobials. However, this comes with the caveat that not all antimicrobials tested in this study may be compatible with the D300e approach.

AST methods must be versatile so that minimal disruption to laboratory workflow occurs when introducing changes, such as including novel antimicrobials approved for clinical use, breakpoint revisions, as well as accommodating institutional prescribing habits (i.e. combination therapies) or requests for additional testing^25,26^. The D300e approach to create BMD testing panels has several limitations. While the instrument has a small footprint, the dispense cassettes used by the D300e (both T8+ and D4+) are single-use and the number of microplates that can be filled by a single cassette is restricted. Using the plate layout and antimicrobial concentrations described in this study (6 antimicrobials per plate with 9 concentrations each), dispensing was limited to 4, 96-well microtitre plates per cassette to ensure optimal dispense dynamics. This means that a large number of cassettes would be needed to perform daily routine testing. Despite several years between studies, the costs of our D300e instrument and consumables were unchanged from those reported by Smith and Kirby (2016). The requirement of a surfactant (i.e. polysorbate P-20, DMSO or other) for accurate dispensing suggests that surfactant-antibiotic compatibility and toxicity screens would need to be performed with all antimicrobials and test organisms prior to implementing such testing. We used polysorbate P-20 as our test surfactant as it has been examined previously^5^. It is possible that other surfactants may be better suited for dispensing some of the antibiotics tested here, such as the 0.1% Triton X-100 solution used by Ransom et al.^9^; however, this would require additional validation.

In summary, the D300e Digital Dispenser is potentially appealing as a rapid, reproducible and customizable alternative for creating BMD AST panels and performing BMD testing outside of reference centres. Its applications in clinical laboratories could include but are not limited to: routine AST of primary pathogens, AST of organisms with known incompatibilities with automated testing systems, or for synergy testing, as multiple antimicrobial agents can be dispensed into individual microplate wells^8,27^. Additional studies are required to further assess its potential role in this setting and to determine the optimal dispensing conditions for different organism and antimicrobial combinations.

## Supplementary Information

Supplementary Information.
