# Supplementary material for: Evaluation of digital dispense-assisted broth microdilution antimicrobial susceptibility testing for Pseudomonas aeruginosa isolates
Source: Sci Rep. 2021 Apr 28;11:9157. doi: 10.1038/s41598-021-88423-0 (PMC8080699; doi:10.1038/s41598-021-88423-0)
Supplement: Supplementary file 1 — Supplementary Information. [file 41598_2021_88423_MOESM1_ESM.pdf]

Supplementary information

Evaluation of digital dispense-assisted broth microdilution antimicrobial susceptibility testing for *Pseudomonas aeruginosa* isolates

Shawn T Clark, Patrick J Stapleton, Pauline W Wang, Yvonne CW Yau, Valerie J Waters, David M. Hwang, David S Guttman

This document contains the following supplementary information:

Figure S1
Figure S2
Table S1
Table S2
Table S3

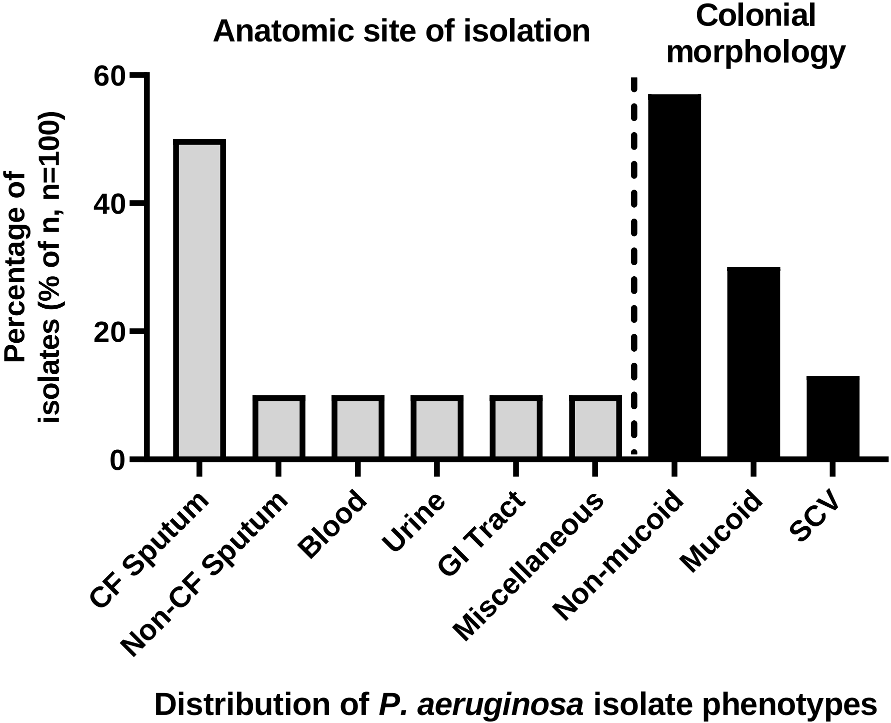
Anatomic site of isolation
Colonial morphology
Percentage of isolates (% of n, n=100)
60
40
20
0
CF Sputum
Non-CF Sputum
Blood
Urine
GI Tract
Miscellaneous
Non-mucoid
Mucoid
SCV
Distribution of P. aeruginosa isolate phenotypes

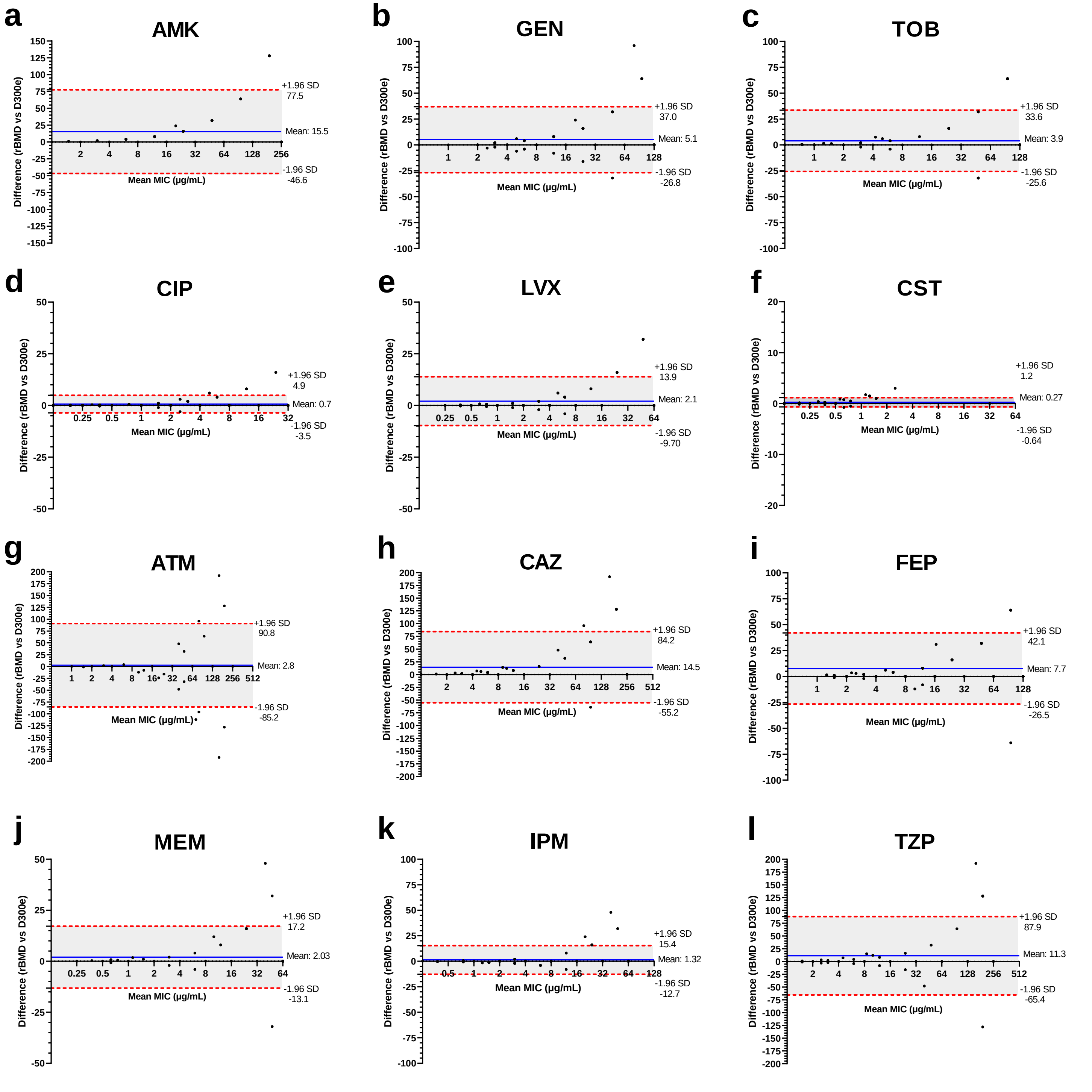

Figure S2. Bland-Altman analysis comparing the two broth microdilution methods (rBMD vs D300e) for antimicrobial susceptibility testing of a) amikacin, b) gentamicin, c) tobramycin, d) ciprofloxacin, e) levofloxacin, f) colistin, g) aztreonam, h) ceftazidime, i) cefepime, j) meropenem, k) imipenem, l) piperacillin-tazobactam. The solid blue line in each plot indicates the mean difference in MIC value while the hatched red lines indicate the ±1.96 times the standard deviation.

Table S1. *P. aeruginosa* isolates used in this study

			Broth microdilution susceptibility profile											
			Aminoglycoside			Fluoroquinolone		Polymyxin	β-lactam or β-lactam-β-lactamase inhibitor					
Isolate ID	Origin	Morphotype	AMK	GEN	TOB	CIP	LVX	CST	ATM	CAZ	FEP	MEM	IPM	TZP
PedCFSputum-1	CF Sputum - pediatric	Non-mucoid	R	R	R	I	I	S	R	R	R	R	I	S
PedCFSputum-2	CF Sputum - pediatric	Non-mucoid	R	R	R	I	S	S	R	R	R	R	R	R
PedCFSputum-3	CF Sputum - pediatric	Non-mucoid	I	R	S	S	S	S	S	S	I	S	S	S
PedCFSputum-4	CF Sputum - pediatric	Non-mucoid	I	R	R	R	R	S	R	R	R	R	R	R
PedCFSputum-5	CF Sputum - pediatric	Non-mucoid	S	R	S	S	S	S	I	S	I	R	R	S
PedCFSputum-6	CF Sputum - pediatric	Non-mucoid	I	R	R	I	S	S	S	S	R	S	S	S
PedCFSputum-7	CF Sputum - pediatric	Non-mucoid	S	R	R	S	S	S	I	S	S	S	S	S
PedCFSputum-8	CF Sputum - pediatric	Non-mucoid	I	R	S	R	R	S	S	S	S	S	S	S
PedCFSputum-9	CF Sputum - pediatric	Non-mucoid	I	R	R	R	R	S	R	R	R	R	R	R
PedCFSputum-10	CF Sputum - pediatric	Non-mucoid	S	R	R	R	R	S	I	R	R	R	R	S
PedCFSputum-11	CF Sputum - pediatric	Mucoid	S	I	S	I	I	S	S	S	S	S	I	S
PedCFSputum-12	CF Sputum - pediatric	Mucoid	S	I	S	I	R	S	S	S	S	S	S	S
PedCFSputum-13	CF Sputum - pediatric	Mucoid	R	R	I	S	S	S	S	S	S	S	S	S
PedCFSputum-14	CF Sputum - pediatric	Mucoid	R	R	R	R	R	S	S	S	S	S	S	S
PedCFSputum-15	CF Sputum - pediatric	Mucoid	R	R	I	I	I	S	S	S	R	S	S	S
PedCFSputum-16	CF Sputum - pediatric	Mucoid	S	R	R	R	R	S	R	R	R	R	R	R
PedCFSputum-17	CF Sputum - pediatric	Mucoid	I	R	S	S	S	S	S	S	S	S	S	S
PedCFSputum-18	CF Sputum - pediatric	Mucoid	S	S	S	S	S	S	S	S	S	S	S	S
PedCFSputum-19	CF Sputum - pediatric	Mucoid	S	I	S	I	I	S	R	S	I	S	S	I
PedCFSputum-20	CF Sputum - pediatric	Mucoid	S	I	S	I	I	S	R	S	I	S	S	I
PedCFSputum-21	CF Sputum - pediatric	SCV	S	R	R	R	R	R	S	S	S	S	S	S
PedCFSputum-22	CF Sputum - pediatric	SCV	R	R	R	I	R	S	R	I	R	S	I	S
PedCFSputum-23	CF Sputum - pediatric	SCV	R	R	S	S	S	S	S	S	S	S	S	S
PedCFSputum-24	CF Sputum - pediatric	SCV	R	R	I	R	R	S	S	S	R	S	S	S
PedCFSputum-25	CF Sputum - pediatric	SCV	R	R	I	R	R	S	S	S	I	S	S	S
AdultCFSputum-1	CF sputum - adult	Non-mucoid	R	R	I	R	R	S	S	S	S	S	R	S
AdultCFSputum-2	CF sputum - adult	Non-mucoid	R	R	R	I	S	S	R	R	R	I	I	R
AdultCFSputum-3	CF sputum - adult	Non-mucoid	S	S	S	R	R	S	R	I	R	I	R	I
AdultCFSputum-4	CF sputum - adult	Non-mucoid	R	R	R	R	R	S	R	R	R	S	S	I
AdultCFSputum-5	CF sputum - adult	Non-mucoid	R	R	R	R	R	S	R	R	R	S	I	R
AdultCFSputum-6	CF sputum - adult	Mucoid	I	S	S	S	S	S	S	S	S	S	S	S
AdultCFSputum-7	CF sputum - adult	Non-mucoid	R	R	I	I	I	S	S	S	R	S	S	S
AdultCFSputum-8	CF sputum - adult	Non-mucoid	R	R	R	R	R	S	S	S	R	I	R	S
AdultCFSputum-9	CF sputum - adult	Non-mucoid	S	S	S	S	S	S	I	I	S	S	S	S
AdultCFSputum-10	CF sputum - adult	Non-mucoid	R	R	R	R	R	S	R	R	R	R	R	R
AdultCFSputum-11	CF sputum - adult	Mucoid	I	R	S	S	S	S	S	S	S	S	S	S
AdultCFSputum-12	CF sputum - adult	Mucoid	R	R	S	I	I	S	R	R	R	R	R	S
AdultCFSputum-13	CF sputum - adult	Mucoid	S	S	S	S	S	S	R	I	S	S	I	I
AdultCFSputum-14	CF sputum - adult	Mucoid	R	R	R	R	R	R	R	R	R	R	R	R
AdultCFSputum-15	CF sputum - adult	Mucoid	R	R	R	R	R	S	R	R	R	R	R	R
AdultCFSputum-16	CF sputum - adult	Mucoid	R	R	R	R	R	S	R	R	R	S	R	S
AdultCFSputum-17	CF sputum - adult	Mucoid	S	R	R	I	I	S	S	S	I	S	S	S
AdultCFSputum-18	CF sputum - adult	Mucoid	S	S	S	I	I	S	S	S	R	S	S	S
AdultCFSputum-19	CF sputum - adult	Mucoid	S	R	R	I	R	S	R	S	R	S	I	S
AdultCFSputum-20	CF sputum - adult	Mucoid	R	R	S	I	R	S	R	R	I	S	S	I
AdultCFSputum-21	CF sputum - adult	SCV	I	R	I	R	R	S	I	R	I	S	S	I
AdultCFSputum-22	CF sputum - adult	SCV	R	R	R	I	I	S	S	S	S	S	I	S
AdultCFSputum-23	CF sputum - adult	SCV	I	R	S	R	R	S	R	R	S	R	R	R
AdultCFSputum-24	CF sputum - adult	SCV	R	R	R	R	R	S	R	R	R	R	R	R
AdultCFSputum-25	CF sputum - adult	SCV	S	S	S	S	S	S	S	S	I	S	S	S
NonCFSputum-1	Sputum (Non-CF)	Non-mucoid	S	R	R	R	R	S	S	S	S	S	S	S
NonCFSputum-2	Sputum (Non-CF)	Mucoid	I	S	S	R	R	S	S	S	S	S	S	S
NonCFSputum-3	Sputum (Non-CF)	Mucoid	S	I	S	R	R	S	R	R	R	S	I	R
NonCFSputum-4	Sputum (Non-CF)	Non-mucoid	I	I	S	S	S	S	S	S	I	S	S	S
NonCFSputum-5	Sputum (Non-CF)	Non-mucoid	R	R	R	R	R	S	R	R	R	I	I	R
NonCFSputum-6	Sputum (Non-CF)	Mucoid	I	S	S	S	S	S	S	S	I	S	S	S
NonCFSputum-7	Sputum (Non-CF)	Mucoid	S	S	S	R	R	S	S	S	S	S	S	S
NonCFSputum-8	Sputum (Non-CF)	Mucoid	S	S	S	S	S	S	R	R	R	I	R	R
NonCFSputum-9	Sputum (Non-CF)	Mucoid	I	R	I	R	S	S	S	S	S	S	I	S
NonCFSputum-10	Sputum (Non-CF)	SCV	S	S	S	I	I	S	S	S	S	S	S	S
Sterilesite-1	Blood	Non-mucoid	S	S	S	S	S	S	S	I	S	I	R	S
Sterilesite-2	Blood	Non-mucoid	S	S	S	S	S	S	I	S	S	I	R	S
Sterilesite-3	Blood	Non-mucoid	S	S	S	S	S	S	S	S	S	S	S	S
Sterilesite-4	Blood	SCV	S	S	S	S	S	S	I	S	S	S	S	S
Sterilesite-5	Blood	SCV	R	R	I	R	R	S	R	R	R	R	R	R
Sterilesite-6	Blood	Mucoid	S	S	S	S	I	S	I	S	S	S	S	S
Sterilesite-7	Blood	Mucoid	S	S	S	R	R	S	R	I	R	R	R	S
Sterilesite-8	Blood	Mucoid	S	S	S	S	S	S	I	S	S	S	S	S
Sterilesite-9	Blood	Non-mucoid	S	S	S	S	S	S	I	S	S	S	S	S
Sterilesite-10	Blood	Non-mucoid	S	S	S	S	S	S	S	S	S	S	S	S
Urinary-1	Urine	Non-mucoid	R	R	R	R	R	S	R	R	R	R	R	I
Urinary-2	Urine	Non-mucoid	R	R	R	R	R	S	R	R	R	R	R	R
Urinary-3	Urine	Non-mucoid	I	R	R	R	R	S	I	R	R	R	R	R
Urinary-4	Urine	Non-mucoid	S	R	R	R	R	S	R	R	R	R	R	R
Urinary-5	Urine	Non-mucoid	S	S	S	I	R	S	R	I	S	S	S	I
Urinary-6	Urine	Non-mucoid	S	S	S	S	S	S	S	S	S	S	I	S
Urinary-7	Urine	Non-mucoid	S	S	S	R	R	S	I	S	S	I	S	S
Urinary-8	Urine	Non-mucoid	S	R	R	R	R	S	R	R	R	R	R	R
Urinary-9	Urine	Non-mucoid	R	R	R	R	R	S	R	R	R	R	R	I
Urinary-10	Urine	Non-mucoid	S	S	S	S	S	S	S	S	S	R	R	S
LowerGI-1	Rectal swab	Non-mucoid	S	S	S	S	I	S	S	S	S	S	S	S
LowerGI-2	Rectal swab	Non-mucoid	S	S	S	S	S	S	I	S	S	S	I	S
LowerGI-3	Rectal swab	Non-mucoid	S	I	S	S	S	S	I	S	S	S	S	S
LowerGI-4	Rectal swab	Non-mucoid	R	R	R	R	R	S	R	R	R	R	R	R
LowerGI-5	Rectal swab	Non-mucoid	S	S	S	S	S	S	R	S	S	S	S	S
LowerGI-6	Rectal swab	Non-mucoid	S	S	S	S	S	S	I	S	S	S	I	S
LowerGI-7	Rectal swab	Non-mucoid	S	S	S	S	S	S	I	S	S	S	S	S
LowerGI-8	Rectal swab	Non-mucoid	R	R	R	R	R	S	R	R	R	R	R	R
LowerGI-9	Rectal swab	Non-mucoid	I	R	R	S	S	S	R	R	R	S	I	R
LowerGI-10	Rectal swab	Non-mucoid	I	R	R	R	R	S	R	R	R	R	R	R
Misc-1	Corneal scraping	Non-mucoid	S	S	S	S	S	S	S	S	S	S	S	S
Misc-2	Wound	Non-mucoid	I	I	S	S	S	S	I	S	S	S	S	S
Misc-3	Wound	Non-mucoid	R	R	R	R	R	S	R	R	R	R	R	R
Misc-4	Wound	Non-mucoid	R	R	R	R	R	S	R	I	R	R	R	R
Misc-5	Wound	Non-mucoid	S	S	S	S	S	S	S	S	S	S	S	S
Misc-6	Wound	Non-mucoid	R	R	R	R	R	S	R	R	R	R	R	R
Misc-7	Conjunctiva	Non-mucoid	R	R	R	R	R	S	R	R	I	R	R	R
Misc-8	Eye	Non-mucoid	S	S	S	S	I	S	R	R	R	S	S	R
Misc-9	Conjunctiva	Non-mucoid	S	S	S	S	S	S	I	S	S	R	R	S
Misc-10	Wound	Non-mucoid	S	R	S	R	R	S	R	R	R	R	R	I

Table S2. Antimicrobial susceptibility profiles of *P. aeruginosa* isolates in this study

Antimicrobial	Percent Resistant (%)[a]	D300e		rBMD	
		MIC_{50} (µg/mL)	MIC_{90} (µg/mL)	MIC_{50} (µg/mL)	MIC_{90} (µg/mL)
Aminoglycosides					
AMK	34	16	256	32	128
GEN	58	16	128	16	128
TOB	39	4	128	4	128
β-lactams					
ATM	44	32	128	16	128
CAZ	37	4	256	8	256
FEP	44	16	128	16	128
IPM	37	4	64	4	64
MEM	31	1	64	1	64
TZP	28	8/4	256/4	16/4	256/4
Fluoroquinolones					
CIP	44	1	16	1	16
LVX	48	2	16	2	32
Polymyxin					
CST	2	0.25	0.5	0.5	1

[a] As determined by rBMD testing

Table S3. Replicate testing of *P. aeruginosa* isolates with ME or VME

Susceptibility patterns (μg/mL)[a]

Isolate	Replicate #	AMK						GEN						TOB						CIP					
		D300e		BMD		Log_2 Diff	Cat Error	D300e		BMD		Log_2 Diff	Cat Error	D300e		BMD		Log_2 Diff	Cat Error	D300e		BMD		Log_2 Diff	Cat Error
		MIC	Categ	MIC	Categ			MIC	Categ	MIC	Categ			MIC	Categ	MIC	Categ			MIC	Categ	MIC	Categ		
Misc-1	1	2	S	8	S	-2	No	2	S	8	I	-2	MinE	0.5	S	1	S	-1	No	0.125	S	1	I	-3	MinE
	2	4	S	8	S	-1	No	2	S	4	S	-1	No	1	S	2	S	-1	No	0.125	S	0.25	S	-1	No
	3	4	S	8	S	-1	No	2	S	4	S	-1	No	1	S	1	S	0	No	0.125	S	0.25	S	-1	No
	Consensus MIC	4	S	8	S	-1	No	2	S	4	S	-1	No	1	S	1	S	0	No	0.125	S	0.25	S	-1	No
Misc-8	1	64	R	4	S	4	ME	16	R	4	S	2	ME	8	I	1	S	3	MinE	0.5	S	0.25	S	1	No
	2	8	S	8	S	0	No	8	S	8	S	0	No	1	S	1	S	0	No	0.25	S	0.5	S	-1	No
	3	8	S	8	S	0	No	2	S	2	S	0	No	1	S	2	S	-1	No	0.25	S	0.25	S	0	No
	Consensus MIC	8	S	8	S	0	No	8	S	4	S	1	No	1	S	1	S	0	No	0.25	S	0.25	S	0	No

Isolate	Replicate #	LEV						CST						ATM						CAZ					
		D300e		BMD		Log_2 Diff	Cat Error	D300e		BMD		Log_2 Diff	Cat Error	D300e		BMD		Log_2 Diff	Cat Error	D300e		BMD		Log_2 Diff	Cat Error
		MIC	Categ	MIC	Categ			MIC	Categ	MIC	Categ			MIC	Categ	MIC	Categ			MIC	Categ	MIC	Categ		
Misc-1	1	0.5	S	4	R	-3	VME	0.125	S	2	S	-3	No	32	R	1	S	5	ME	2	S	1	S	1	No
	2	0.5	S	1	S	-1	No	0.25	S	0.5	S	-1	No	32	R	16	I	1	MinE	4	S	4	S	0	No
	3	0.5	S	1	S	-1	No	0.25	S	0.5	S	-1	No	16	I	8	S	1	MinE	4	S	4	S	0	No
	Consensus MIC	0.5	S	1	S	-1	No	0.25	S	0.5	S	-1	No	32	R	8	S	2	ME	4	S	4	S	0	No
Misc-8	1	2	I	0.5	S	2	MinE	8	R	0.25	S	5	ME	4	S	128	R	-5	VME	4	S	128	R	-4	VME
	2	1	S	2	I	-1	MinE	0.25	S	0.5	S	-1	No	64	R	64	R	0	No	64	R	64	R	0	No
	3	0.5	S	2	I	-2	MinE	1	S	0.5	S	1	No	128	R	128	R	0	No	64	R	128	R	-1	No
	Consensus MIC	1	S	2	I	-1	MinE	1	S	0.5	S	-1	No	64	R	128	R	-1	No	64	R	128	R	-1	No

Isolate	Replicate #	FEP						MEM						IPM						TZP					
		D300e		BMD		Log_2 Diff	Cat Error	D300e		BMD		Log_2 Diff	Cat Error	D300e		BMD		Log_2 Diff	Cat Error	D300e		BMD		Log_2 Diff	Cat Error
		MIC	Categ	MIC	Categ			MIC	Categ	MIC	Categ			MIC	Categ	MIC	Categ			MIC	Categ	MIC	Categ		
Misc-1	1	2	S	0.5	S	2	No	1	S	0.25	S	2	No	2	S	0.25	S	3	No	1/4	S	1/4	S	0	No
	2	2	S	2	S	0	No	2	S	2	S	0	No	2	S	2	S	0	No	4/4	S	8/4	S	-1	No
	3	0.5	S	1	S	-1	No	2	S	2	S	0	No	2	S	2	S	0	No	4/4	S	8/4	S	-1	No
	Consensus MIC	2	S	1	S	1	No	2	S	2	S	0	No	2	S	2	S	0	No	4/4	S	8/4	S	-1	No
Misc-8	1	0.5	S	32	R	-6	VME	0.5	S	0.5	S	0	No	0.5	S	1	S	-1	No	2/4	S	128/4	R	-7	VME
	2	32	R	32	R	0	No	1	S	1	S	0	No	2	S	2	S	0	No	128/4	R	128/4	R	0	No
	3	1	S	2	S	-1	No	0.5	S	2	S	-2	No	2	S	2	S	0	No	128/4	R	128/4	R	0	No
	Consensus MIC	1	S	32	R	0	VME	0.5	S	1	S	-1	No	2	S	2	S	0	No	128/4	R	128/4	R	0	No

[a] Determined as per CLSI 2020 interpretive criteria (CLSI, 2020)
